# Basic fibroblast growth factor accelerates myelin debris clearance through activating autophagy to facilitate early peripheral nerve regeneration

**DOI:** 10.1111/jcmm.16274

**Published:** 2021-01-29

**Authors:** Yongsheng Jiang, Jiahong Liang, Rui Li, Yan Peng, JiangLi Huang, Lijiang Huang

**Affiliations:** ^1^ The Affiliated Xiangshan Hospital of Wenzhou Medial University Zhejiang China; ^2^ HangZhou Zhuyangxin Pharmaceutical Co.,LTD Hangzhou Zhejiang China; ^3^ PCFM Lab, GD HPPC Lab School of Chemistry Sun Yat‐sen University Guangzhou China; ^4^ Hangzhou Institute for Food and Drug control Hangzhou Zhejiang China

**Keywords:** autophagy, basic fibroblast growth factor, myelin clearance, peripheral nerve regrowth

## Abstract

The successful removal of damaged myelin sheaths during Wallerian degeneration (WD) is essential for ensuring structural remodelling and functional recovery following traumatic peripheral nerve injury (PNI). Recent studies have established that autophagy involves myelin phagocytosis and cellular homoeostasis, and its disorder impairs myelin clearance. Based on the role of basic fibroblast growth factor (bFGF) on exerting neuroprotection and angiogenesis during nerve tissue regeneration, we now explicitly focus on the issue about whether the therapeutic effect of bFGF on supporting nerve regeneration is closely related to accelerate the autophagic clearance of myelin debris during WD. Using sciatic nerve crushed model, we found that bFGF remarkedly improved axonal outgrowth and nerve reconstruction at the early phase of PNI (14 days after PNI). More importantly, we further observed that bFGF could enhance phagocytic capacity of Schwann cells (SCs) to engulf myelin debris. Additionally, this enhancing effect is accomplished by autophagy activation and the increase of autophagy flux by immunoblotting and immune‐histochemical analyses. Taken together, our data suggest that the action of bFGF on modulating early peripheral nerve regeneration is closely associated with myelin debris removal by SCs, which might result in SC‐mediated autophagy activation, highlighting its insight molecular mechanism as a neuroprotective agent for repairing PNI.

## 1 INTRODUCTION

Traumatic peripheral nerve injury (PNI) is a steadily increasing incidence worldwide characterized by progressive sensory loss, motor disorder, neuropathic pain or a combination of functional disability.^1^, ^2^ Despite mammalian peripheral nervous system (PNS) has an intrinsic regenerative capacity for supporting axonal elongation and myelination, up to now, there is no seeking an available therapeutic strategy for completely restoring damage nerve functional recovery.^3^, ^4^ Researchers and clinicians have been striving to acquire a comprehensive understanding of molecular mechanisms underlying post‐injured neurologic degeneration and regeneration and to develop new therapeutic options for ameliorating nerve regenerative capability.

Following peripheral nerve damage, the lesion region and its distal stump undergo extensive morphological and biochemical changes, including axon degeneration, demyelination and phagocytosis, a process known as Wallerian degeneration (WD). The breakdown of myelin sheaths during WD will generate abundant myelin debris, which seriously affect nerve regeneration and functional recovery.^5^, ^6^ Moreover, the prolonged presence of myelin fragments will act as potent inflammatory stimuli which creates an inhibitory microenvironment to dramatically impair reinnervation of potential peripheral targets.^7^, ^8^ Accordingly, efficient degradation and clearance of axonal and myelin fragments during WD is a prerequisite for successful axonal regeneration and target reinnervation.

Observations in experimental animals with PNI found that both Schwann cells (SCs) and macrophages were respond to myelin clearance, but we still unknown the major contributors. Ghabriel et al initially discovered that SCs themselves broke down the phagocytic myelin fragmentats into small ovoid‐like structures using electron microscopic monitoring.^9^ Other reports also revealed that the mechanistic understanding of SC‐mediated myelin debris clearance was associated with activating the Axl and Mertk pathways following injury.^10^ More importantly, it has been estimated that approximately 40%‐50% residual myelin debris were removed by SCs during the first 5‐7 days after injury.^11^, ^12^ Thus, we deem that concerning the role of myelin debris clearance by SCs and their cellular and molecular mechanisms is possible an essential modulation for triggering axon degeneration and nerve regeneration after traumatic injury to peripheral nerve.

Basic fibroblast growth factor (bFGF) is one of powerful neurotrophic factors secreted by SCs and different neuronal populations. Following peripheral nerve damage, up‐regulation of bFGF mRNA and protein were still not maintain neuronal survival and axonal elongation.^13^ Overexpression of bFGF within SCs via lentivirus transfection was able to facilitate muscular reinnervation and stimulate both motor and sensory neuronal regeneration in the PNS.^14^ bFGF also created a permissive growth environment to exert multiple biological functions, including neuroprotection, neurogenesis and angiogenesis.^15^, ^16^ Previous works in our group had demonstrated that a thermosensitive heparin‐poloxamer hydrogel incorporating bFGF was efficiently facilitating SC proliferation, axonal regeneration and motor function recovery in diabetic rats suffering from PNI.^17^ Collectively, bFGF has been regarded as a promising growth factor for efficiently improving the peripheral nerve repair, but it remained unclear whether bFGF‐induced nerve tissue regeneration is associated with SCs‐mediated myelin phagocytosis. In addition, the potential molecular mechanism of their relationship remains to be elucidated.

Macroautophagy (termed hereafter autophagy) is a ubiquitous cytoprotective process that plays a critical role in maintaining cellular and tissue homeostasis by degrading and recycling of cellular constituents, including damaged organelles, pathological proteins and dysfunctional macromolecules. Autophagy regulates several physiological and pathological processes, such as myelin degradation, myelinating development, nerve regeneration and neuropathic pain, through a lysosomal degradative process. Gomez‐Sanchez et al explained that the cellular mechanism involving damaged myelin degradation during WD was closely linked with activation of autophagy within SCs.^18^ Conditional knockout critical autophagy gene‐atg 7 resulted in a delayed myelin clearance and thickened axonal cytoplasm, which seriously affected SC structural plasticity during myelination.^19^, ^20^ Moreover, administration of the autophagy inducer rapamycin significantly reduced inflammatory and immune reaction, leading to neuropathic pain amelioration.^21^ Additionally, accumulating evidence had demonstrated that up‐regulation of autophagy level was contributed to microtubule stabilization, nerve regeneration and motor function recovery.^22^, ^23^ These convincing outcome indicate that modulation of autophagy reaction within SCs during WD possibly provides an important cellular protecting mechanism for guiding the degradation of damaged intracellular components to facilitate nerve regeneration and functional recovery after PNI.

Here, we initially identify the role of bFGF in neuroprotection and neuroregeneration at the early phage of nerve recovery. Moreover, we demonstrated for the first time that the capacity of bFGF for promoting early axon growth was closely related to its remarkable removal of degenerated myelin debris attributed by SCs. Finally, we further elucidated the molecular mechanism of bFGF on eliminating myelin debris are probably mediated by activation of autophagy within SCs. The current study set out to determine how bFGF guides SCs uptake of myelin debris to facilitate early nerve regeneration.

## 2 MATERIALS AND METHODS

### 2.1 Reagents and antibodies

bFGF lyophilized powder (BFF‐H4117) was purchased from ACRO Biosystems (Beijing, China). Haematoxylin and eosin kit (H&E, C0105), 4′6‐diamidino‐2‐phenylindole‐dihydrochloride (DAPI, C1006), the Micro BCA (Bicinchoninic acid) Protein Assay Kit (P0010), RIPA lysis buffer and PMSF proteinase inhibitor were obtained from Beycotime Biotechnology (Shanghai, China). Masson's trichrome staining kit (Masson, G1340) and Triton X‐100 (T8200) were purchased from Solarbio (Beijing, China). TaKaRa MiniBEST Universal RNA Extraction Kit (9767), PrimeScript RT Master Mix (RR036Q) and SuperPlex™ Premix (638543) were obtained from Takara Bio Inc (Shiga, Japan).

The primary and secondary, used in this study, were listed as following: rabbit polyclonal anti‐beclin‐1 (ab62557, 1:500; Abcam, Shanghai, China), rabbit polyclonal anti‐LC3 (ab128025, 1:1000; Abcam), mouse monoclonal anti‐MBP (Myelin basic protein) (ab62631, 1:300; Abcam), rabbit polyclonal anti‐MPZ (Myelin protein zero) (ab31851, 1:500; Abcam), mouse monoclonal anti‐GFAP (Glial fibrillary acidic protein) (ab10062, 1:300; Abcam), chicken polyclonal anti‐NF‐200 (ab4680, 1:5000; Abcam), rabbit polyclonal anti‐ATG7 (BS6046, 1:500; Bioworld, Nanjing, China), rabbit polyclonal anti‐ATG5 (AP6026, 1:500; Bioworld), rabbit monoclonal *p*‐TFEB (Transcription factor EB) (#37681, 1:1000; CST), goat polyclonal anti‐TFEB (ab2636, 1:1000; Abcam), mouse monoclonal GAPDH (5174T, 1:10 000; CST), goat anti‐mouse IgG‐HRP secondary antibody (BS12478, 1:10 000; Bioworld), Goat anti‐rabbit (H + L) HRP secondary antibody (BS13278, 10 000; Bioworld), donkey anti‐rabbit Alexa Fluor 488‐conjugated secondary antibody, donkey anti‐mouse Alexa Fluor 647‐conjugated secondary antibody (A32787, 1:2000; Thermo Fisher scientific), goat anti‐chicken Alexa Fluor 594‐conjugated secondary antibody (ab150176, 1:1500; Abcam).

### 2.2 Nerve crush injury model and drug administration

All experimental protocols and animal handling procedures were conducted according to the standards of the Animal Care and Use Committee of Sun Yat‐sen University, following the guidelines of the National Institutes of Health Guide for the Care and Use of Laboratory Animals. Male SD rats (200‐220 g), supplied by the Experimental Animal Center of Sun Yat‐sen University, were raised in different cages at room temperature (25°C) for 1 week prior to surgery to adjust to the standardized laboratory environment.

The operation of sciatic nerve crush injury model was according to the previously described.^24^ Briefly, after sterilization with 3% pentobarbital sodium (50 mg/kg), the right sciatic nerve was exposed under the microscope and completely crushed with a pair of vascular clips (2 mm interval, Oscar, Shanghai, China) with 30 g for 2 minutes at 7 mm above the bifurcation. The incision was then closed and sutured with 4‐0 stitches, followed by administering cefazolin sodium (50 mg/kg, i.p.) once daily for consecutive 7 days.

After the surgery, the animals were then randomly divided into three groups: sham group, PNI group and PNI + bFGF group. Each group contained eight rats. For the PNI + bFGF group, the injured sciatic nerve was rapidly treated with 0.1 mL bFGF solution (30 μg/mL)^25^ via orthotopic injection once daily for 5 days. Other groups, including sham and PNI group, were received an equal volume of saline. The animals of the sham group underwent the same procedures, except for the nerve crushing. Observers were blinded to treatment and evaluation.

### 2.3 Tissue preparation

At 5 and 14 days post‐surgery, animals were killed via perfusing with cold normal saline to the left ventricle. After removed blood, 2‐cm‐long segments of sciatic nerves containing the lesion site were dissected and harvested in an Eppendorf tube (2 mL) for further experiments. For morphological analysis, the collected sciatic nerves were post‐fixed in 4% paraformaldehyde (PFA) overnight and then dehydrated with gradient grade ethanol, embedded in paraffin. Lastly, the specimens were cut into thin cross sections of 5 μm thickness with a HM 340E rotary microtome (LEICA, Wetzlar, Germany) and mounted on poly‐L‐lysine‐coated slides. For immunofluorescence, the nerve segments were kept in 4% PFA for 4 hours, dehydrated and embedded in optimal cutting temperature, then cut into 10 μm thick cross sections or longitudinal sections on an ultramicrotome (MT‐XL; RMC Inc, Hertfordshire, USA) and mounted directly on glass slides. For Western blotting and reverse transcription‐polymerase chain reaction analyses, fresh nerve tissues were immediately stored in liquid nitrogen.

### 2.4 Histological analysis

The prepared sections were subjected to H&E staining kit or Masson's trichrome staining kit based on the manufacture's protocol. Briefly, after drying at 56°C for 30 minutes, sections were immersed in xylene and rehydrated by a series of ethanol solution. Afterwards, the sections were added different staining reagents. For H&E staining, the clean samples were submerged in haematoxylin for 5 minutes, followed by stained with eosin for 10 minutes. For Masson's trichrome staining, the clean sections were stained the cell nuclei using haematoxylin for 5 minutes and added Ponceau acid fuchsin solution to cover whole tissues for 5 minutes, followed by 30 seconds in 1% phosphomolybdic acid solution and 1 minutes in aniline blue reagent. Finally, slides were mounted with neutral resin and coverslipped. The stained sections were visualized and imaged using a Nikon ECLIPSE Ti microscope (Nikon, Tokyo, Japan).

### 2.5 Immunofluorescence staining

Cultured cells or tissue sections were fixed at room temperature in 4% PFA for 30 minutes, followed by incubation with 0.1% triton X‐100 for 15 minutes and blocked with 5% bovine serum albumin for 30 minutes. Then, cells or tissue samples were incubated with primary antibodies against GFAP, LC3 and MPZ overnight at 4°C. After washing, these samples were incubated with Alexa FluoAlexa Fluor‐conjugated secondary antibodies at room temperature for 1 hour, followed by labelling by DAPI (1 mg/mL) for 5 minutes. Finally, the fluorescence images were captured using laser confocal scanning fluorescence microscopy (Nikon, A1 PLUS, Tokyo, Japan) and the fluorescent intensity was quantified with ImageJ software (National Institutes of Health, Bethesda, MD, USA).

### 2.6 Immunoblotting analysis

The sciatic nerve samples were harvested and homogenized with RIPA lysis buffer containing 1% PMSF on ice for 30 minutes. After 12 000 *g* centrifugation for 15 minutes at 4°C, the protein concentration in the supernatant was quantified using BCA reagents. Equal amounts of total protein (80 μg) were resolved by SDS (Sodium dodecyl sulfate)‐polyacrylamide gel electrophoresis and then transferred onto a PVDF (Polyvinylidene fluoride) membrane. Membranes were blocked with skimmed milk and probed with primary antibodies (including beclin‐1, LC3, ATG5, ATG7, *p*‐TFEB and TFEB) overnight at 4°C. The next day, the membranes were incubated with secondary antibody conjugated with HRP (Horseradish peroxidase) for 1 hour at room temperature. Protein bands were detected using an enhanced chemiluminescence kit, and the band densities were quantified using ImageJ software.

### 2.7 Reverse transcription‐polymerase chain reaction

Total RNA was extracted from the sciatic nerves using the TaKaRa MiniBEST Universal RNA Extraction Kit according to the manufacturer's protocol. Then, the cDNA of each sample was amplified via reverse transcription of total RNA (2 μg) using PrimeScript RT Master Mix kit. Lastly, samples were run in parallel with each primer set in real‐time PCR with SuperPlex™ Premix and relative fold changes were calculated using the 2^−ΔΔCT^ method. Primers are shown in Table 1. β‐actin was utilized as internal control.

**TABLE 1:** Primers used for RT‐PCR in this study

Gene	Prime sequence	Product size (bp)	Serial number
β‐actin	F: GCAAGTGCTTCTAGGCGGACTG R: CTGCTGTCACCTTCACCGTTCC	195	NM_001101683.1
MBP	F: AGTCCGACGAGCTACAGACCATC R: TACTTGGAGCCGTGCCTCTGG	106	XM_017338987.1
MPZ	F: TCATCGAGATGGAGCTACGGAAGG R: GGCGTTCTTGAGGCTGGTTCTG	89	XM_008264187.2
Erg2	F: CGGGATCCAGTGTTAGCAAGCGCA R: GGAATTCCCAATTGTAATAGCTTTC	668	XM_017338174.1
MAP‐2	F: GATCTGGCAGGCACAAGGTCAAG R: TTCCTCAACTACCGTCTCCGATGG	96	XM_017343068.1
GAP‐43	F: GAAGGCGAGGCTGACCAAGAAC R: AGACGTGAGCAGGACAGGAAGG	141	XM_008266894.2

Abbreviation: RT‐PCR, reverse transcription‐polymerase chain reaction.

### 2.8 Myelin debris preparation

The preparation of myelin debris was accordingly to the following procedures described elsewhere.^26^ Briefly, the exposed sciatic nerves from adult rat were isolated and carefully detached outer membrane using ophthalmic forceps. Then, the inner nerve fibres were cut into small pieces with the length of approximately 3‐4 mm. Next, the shredded samples were collected into a 2 ml Eppendorf tube which contained 300 μL sucrose solution (0.27 M), followed by adequate homogenization using a homogenizer (PRO‐200, Amazing Life, Shanghai, China). After centrifugation at 16 099 g/min for 10 minutes, myelin debris at the bottom of tube were resuspended in PBS (Phosphate buffered solution) to a final concentration of 1 mg/mL.

### 2.9 Myelin debris clearance assay

RSC96 cells (A rat SC line) were provided from ScienCell Research Laboratories. After passaging for three times, they were seeded and cultured on six‐well plates with a density of 5 × 10^6^ cells per well for 24 hours, followed by supplementing 100 μL fresh myelin fractions solution to the medium. After incubating at 37°C/5% CO_2_ for another 24 hours, the medium was added with/without bFGF solution to ensure its concentration in the medium at 100 ng/mL^17^ and cultured for another 24 hours (recorded as 48 hours). Lastly, the unphagocytosed myelin debris were clear away and the RSC 96 cells, including endocytic myelin, were harvested at the indicated time‐points and stained with MBP. We regarded the cell medium adding only myelin debris as the control group. The cell medium containing myelin debris and bFGF was taken as the bFGF group. The total MBP^+^ fluorescent intensity in each group at different time was quantified using ImageJ software and normalized by the control group at 0 time‐point.

### 2.10 Myelin debris engulf analysis

We used primary human SCs (HSCs, Cat# 1700; ScienCell, CA, Carlsbad, USA) to conduct this experiment. After seeding in six‐well plates at the density of 1.00 × 10^6^ cells/well for 24 hours, HSCs were washed three times in PBS and replaced with the fresh culture medium which added 800 μg/mL pHrodo™ Red, succinimidyl ester (P36600, pHrodo; Thermo Fisher Scientific)–labelled myelin debris plus the bFGF solution at doses of 100 ng/mL for incubating at 37°C/5% CO_2_ for additional 6, 12, 18 and 24 hours, respectively. The process of myelin conjugation with pHrodo and drug concentration was referred to the previous research.^17^, ^27^ Myelin uptake was observed under a Nikon ECLIPSE Ti microscope (Nikon, Japan) and quantified with ImageJ.

### 2.11 Monitoring autophagic flow

RSC96 cells were cultured in Dulbecco's modified eagle medium supplemented with 10% foetal bovine serum and 1% streptomycin/penicillin. After grow approximately 70% confluent, cells were infected with tandem fluorescent mRFP‐GFP‐LC3 adenoviral vectors (HanBio, Shanghai, China) for 24 hours. Then, the old culture medium was replaced with fresh medium supplemented with 10 μg/mL^28^ myelin extracts alone or in combination with bFGF (100 ng/mL). Meanwhile, the cells were placed in a humidified incubator with 5% CO_2_ and incubated in this condition for another 4 hours. Lastly, the living cells were imaged for the expression of autophagosomes and autolysosomes under confocal microscopy (Carl Zeiss, Zena, Germany). This fluorescent reporter can efficiently monitor LC3 flux based on GFP (pKa_GFP_ = 6.0) degrades in acidic environment whereas mRFP (pKa_mRFP_ = 4.5) fluorescence persists under this environment. Thus, the colocalization of both mRFP and GFP fluorescence signals (yellow puncta, ie RFP^+^GFP^+^) indicate autophagosomes, whereas only red puncta without GFP fluorescence emission indicate autophagolysosomes (ie RFP^+^GFP^−^). For quantification, an observer without knowledge of the experimental design blindly selected 50 cells from each group. The yellow and red‐only puncta can be automatically counted by Image‐Pro Plus software as per well established methods.

### 2.12 Statistical analysis

All data were expressed as mean ± SEM. Comparisons within multiple groups were made by one‐way ANOVA followed by Tukey's post hoc test (parametric). Two‐tailed unpaired Student's *t* test was used for significant differences between two groups. All statistical analyses were performed using GraphPad Prism 8 software (GraphPad Software, Inc, San Diego, CA, USA). Significance levels were indicated as follows: *P < 0.05, **P < 0.01.

## 3 RESULTS

### 3.1 bFGF supports nerve regeneration during early post‐injury

**FIGURE 1:**
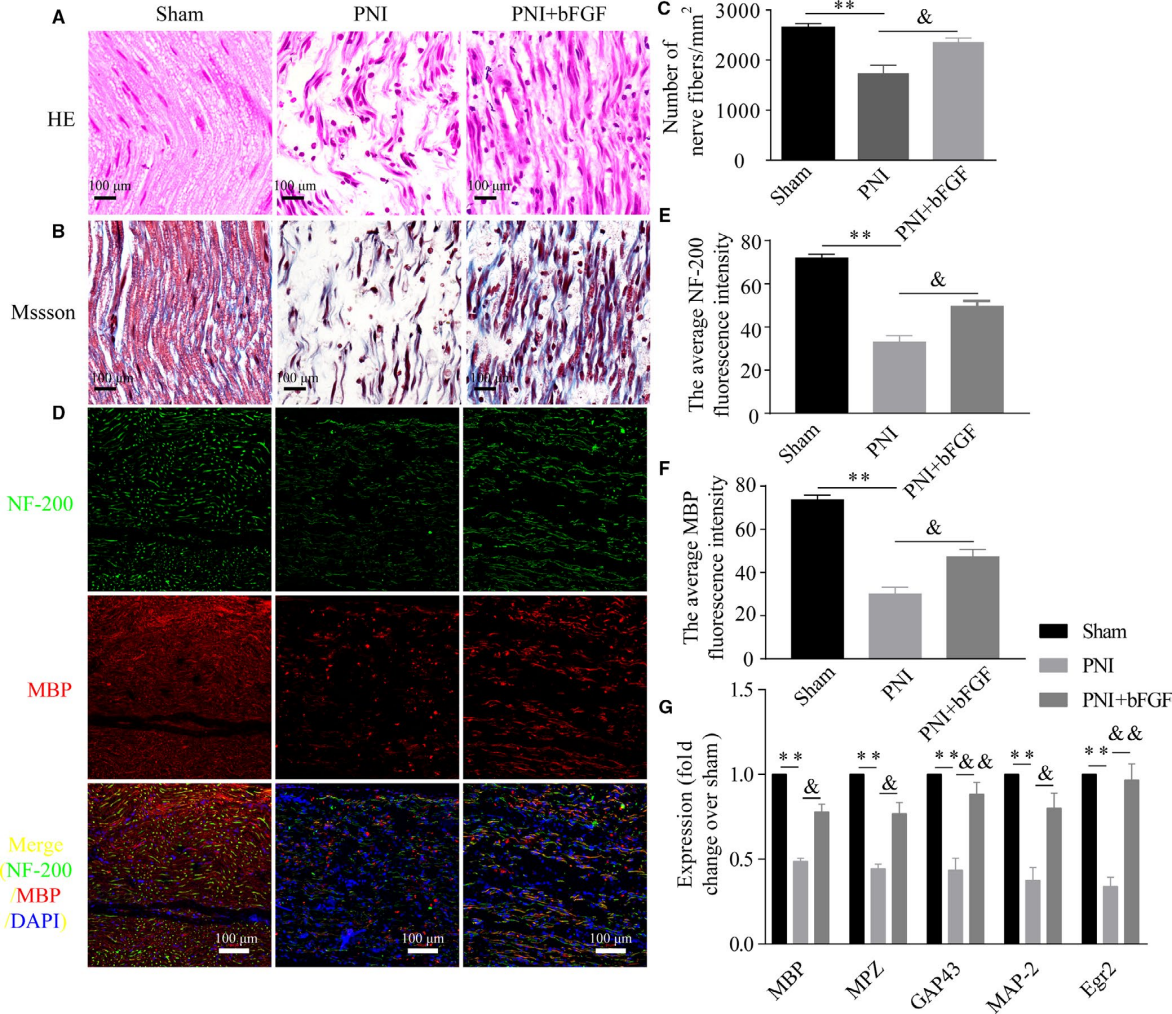
Histological analysis of sciatic nerves at 2 wk of injection of bFGF. A and B, Midportion of the regenerated nerves at 2 wk after surgery were stained with H&E (scale bar = 100 μm) or toluidine blue (scale bar = 100 μm), respectively. C, Quantification of the number of nerve fibres per mm^2^ from (A). D, Confocal laser‐scanning micrographs of sprouting axons and regenerating myelin immunostained with anti‐NF‐200 (green) and anti‐MBP (red) antibodies. Nucleus was marked with DAPI. scale bar = 100 μm. E and F, Quantitative the average of NF‐200 and MBP fluorescence from (D). G, The mRNA levels of MBP, MPZ, GAP43, MAP‐2 and Egr2 were measured by RT‐PCR. Values are represented as means ± SEM. bFGF, basic fibroblast growth factor; DAPI, 4′6‐diamidino‐2‐phenylindole‐dihydrochloride; H&E, haematoxylin and eosin; RT‐PCR, reverse transcription‐polymerase chain reaction. **P < 0.01 as compared to the control group. ^&^ *P* < 0.05 and ^&&^ *P* < 0.01 as compared to the PNI group

We firstly validated whether exogenous administration of bFGF had a significant function on promoting sciatic nerve regeneration at 14 day after injury. The results of H&E and Masson dye staining showed that the nerve fibres after PNI were scarce and atrophic, but this abnormal morphology was significantly ameliorated when PNI animals receiving bFGF treatment (Figure 1A,B). Quantification result also showed that the regenerative nerve fibres in the PNI + NGF group exhibited a marked increase compared with that in the PNI group (Figure 1C). To further verify this observation, we co‐immunolabelled sciatic nerve sections with NF‐200 (marker for axons) and MBP (staining for myelin sheath). Consistent with the histological analyses, the MBP and NF‐200 immunoreactivity in the PNI + NGF group was significant higher than that of PNI group (Figure 1D‐F). Meanwhile, the expression of myelination and growth‐related genes, including MBP, MPZ, MAG, GAP43 and MAP‐2, was remarkably increased once PNI rats receiving bFGF treatment (Figure 1G). These data indicate that bFGF indeed enhances nerve regeneration and axonal remyelination at the early period post‐injury.

### 3.2 bFGF enhances the degradation of myelin segments by SCs

**FIGURE 2:**
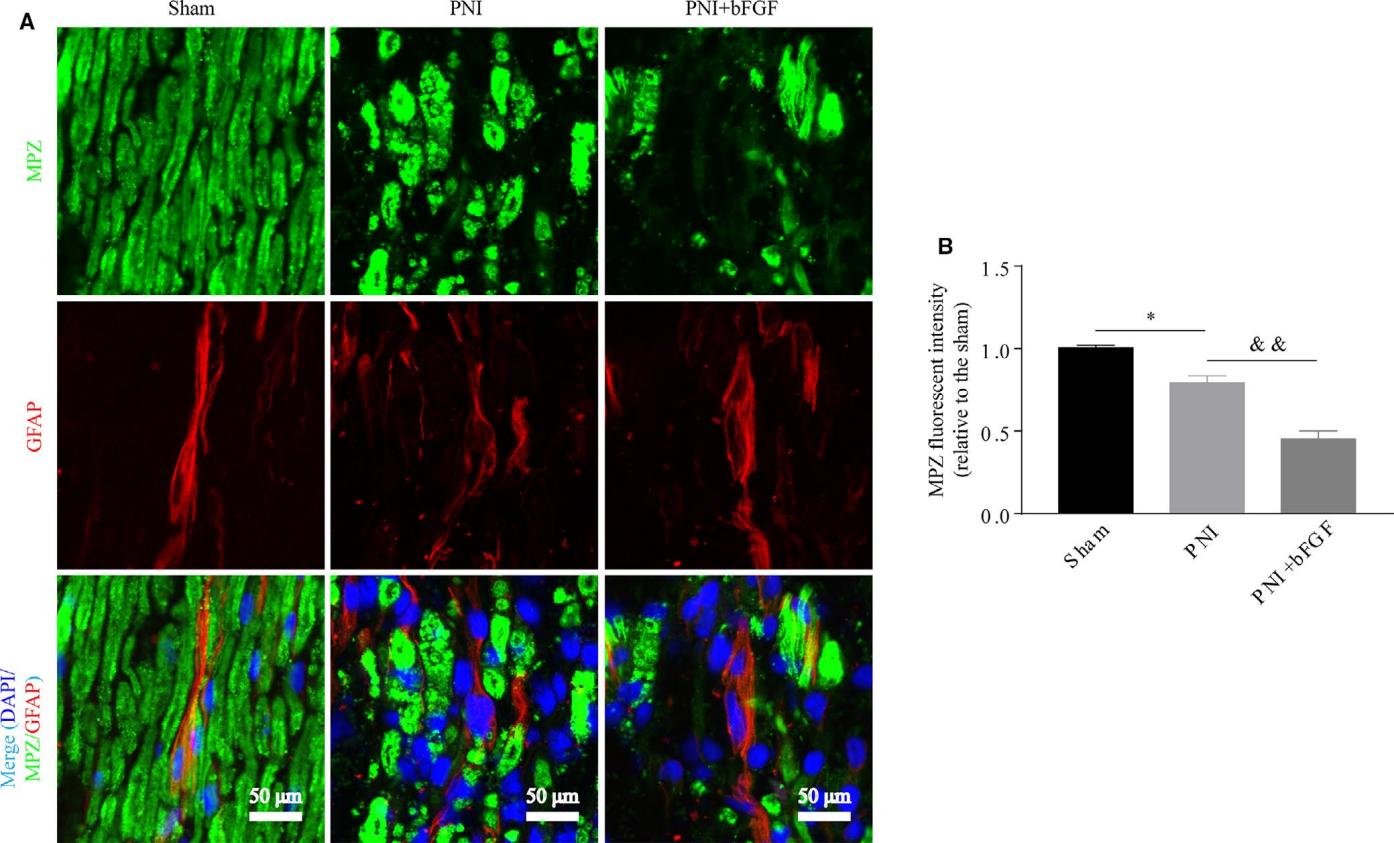
bFGF accelerates myelin debris clearance in PNI rat model at 5 d post‐operation. A, Representative photomicrographs of double immunofluorescence staining for MPZ (green) and GFAP (red) in the sham, PNI and PNI + bFGF groups. Nuclei are stained with DAPI (blue). Scale bar = 50 μm. B, Quantification of the relative MPZ fluorescent intensity from (A). Values are means ± SEM. bFGF, basic fibroblast growth factor; DAPI, 4′6‐diamidino‐2‐phenylindole‐dihydrochloride; PNI, peripheral nerve injury. *P < 0.05 as compared to the control group. ^&&^ *P* < 0.01 as compared to the PNI group

Increasing evidence have confirmed that SC‐mediated myelin debris clearance during WD is essential for successful regeneration of the damaged nerve.^10^, ^18^ Therefore, we investigated whether bFGF‐mediated early nerve restoration was strongly associated with accelerating myelin removal by SCs. As shown in Figure 2A and B, at 5 day post‐surgery, the morphology of myelin sheaths was aligned tightly and regularly in control group. However, large deposit of MPZ‐labelled spots, which was termed as myelin debris, was frequently accumulated in the PNI group and some of them were surrounded within the GFAP‐marked SCs. Surprisingly, such a huge presence of myelin debris became largely reduced after the PNI rats receiving exogenous bFGF.

### 3.3 bFGF promotes SC phagocytosis of myelin

**FIGURE 3:**
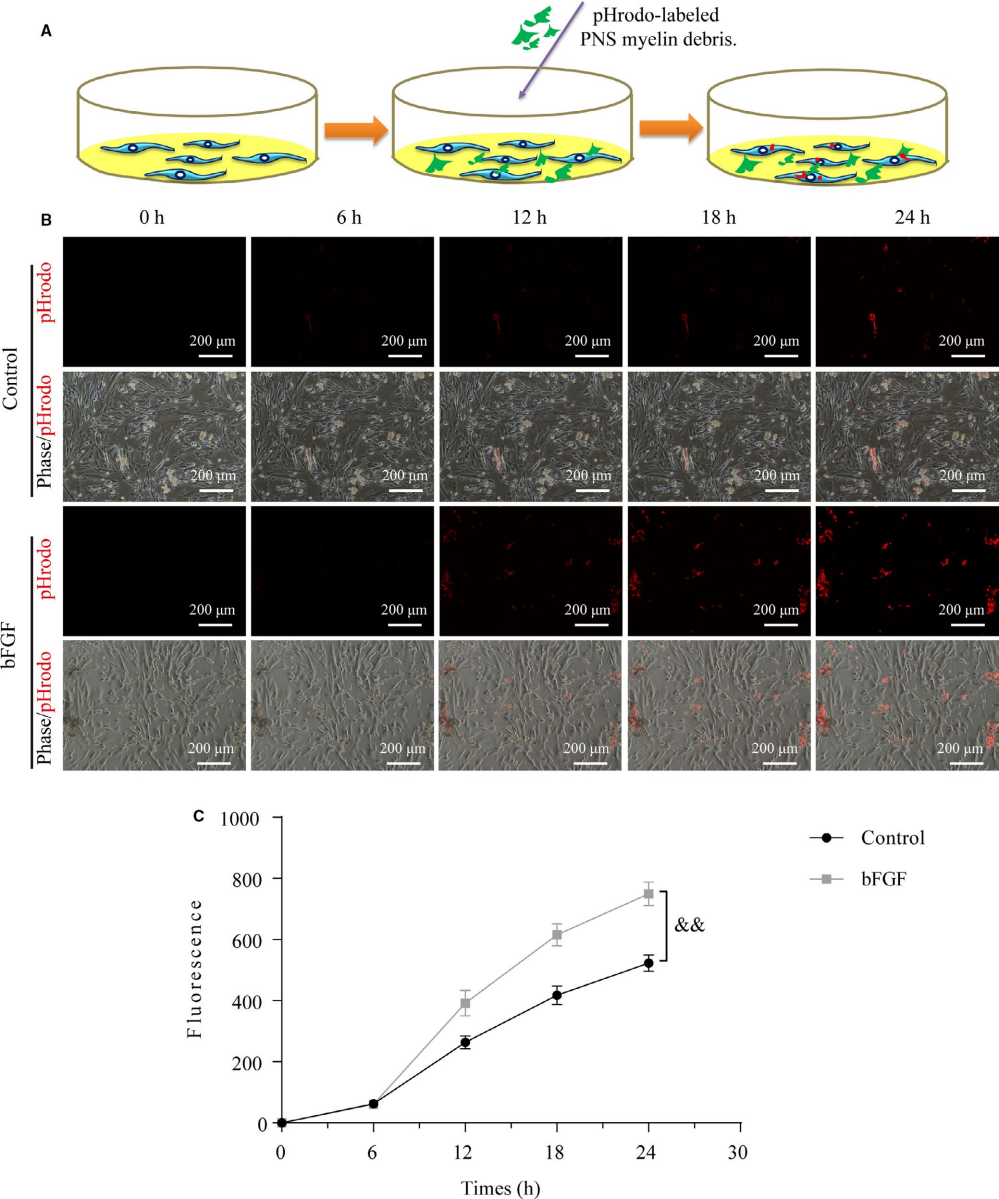
bFGF enhances the action of SCs engulfing myelin debris in vitro. A, Schematic diagram illustrating the specific process of SCs phagocytosis of myelin debris. B, Representative images acquired by a Nikon ECLIPSE Ti microscopy with the phase contrast and fluorescence mode to record primary SCs intake of pHRODO‐labelled myelin at intervals of 6 h. Scale bar = 200 μm. C, Quantification of integrated fluorescence with the treatment indicated above. Data are shown as means ± SEM. bFGF, basic fibroblast growth factor; SC, Schwann cell. ^&&^ *P* < 0.01 as compared to the control group

To further verify this hypothesis, we also added myelin debris to the medium containing with/without bFGF and applied to culture primary SCs (showing in schematic diagram of Figure 3A). Myelin debris were tagged with pH‐sensitive dye pHRODO. This reagent could emit red fluorescent signals in acidic environments, such as SC cytoplasm. Importantly, the stronger labelled debris were engulfed, and the more fluorescent puncta were observed in SCs. Thus, this technique could objectively quantify the number of myelin debris engulfed by SCs. The results of fluorescent and bright field images revealed that the gradual accumulation of myelin debris was occurred in both control and bFGF groups as time elapsed, but the speed of myelin engulfment in bFGF group was faster than that of in control group (Figure 3B). At 24 hour culture, SCs receiving bFGF treatment phagocytosed about twofold more myelin debris than SCs without supplement of bFGF (Figure 3C). These data confirm that SCs are able to swallow myelin debris, which become enhancement once SCs culturing in medium containing bFGF.

However, the fluorescent intensity and distribution of endocytic pHrodo‐labelled myelin debris maybe also affected by the lysosome acidification which caused different PH within cytoplasm or organelle. To eliminate this factor, we firstly detected the PH values of acidic lysosomes within SCs after exposing to pHrodo‐labelled myelin debris + bFGF at indicated time‐points using 2‐(4‐pyridyl)‐5‐((4‐(2‐dimethylaminoethyl‐aminocarbamoyl) methoxy)phenyl)oxazole probe (40768ES50; Yeasen, Shanghai, China).^29^ The result showed that the PH values in acidic lysosomes of SCs within 24 hours engulfment were estimated to be the range of 3.7‐4.5 (Figure S1A). Next, we tested whether the change of PH will affected the fluorescent intensity of pHrodo‐labelled myelin debris. We selected three representative PH values and adjusted the saline's PH with the value of 3.7, 4.1 and 4.5, respectively. Subsequently, we added pHrodo‐labelled myelin debris to the saline for incubating at 37°C for 0.5 hours and imaged with an inverted microscope. As shown in Figure S1B and C, the pHrodo‐labelled myelin debris fluoresced brightly and fluorescence intensity was no statistical difference among these three different PH condition. All of these results indicated the increased pHrodo signal in bFGF‐treating group was mainly because of the increased SC engulfment rather than the enhanced lysosome acidification.

### 3.4 bFGF accelerates the speed of myelin debris removal

**FIGURE 4:**
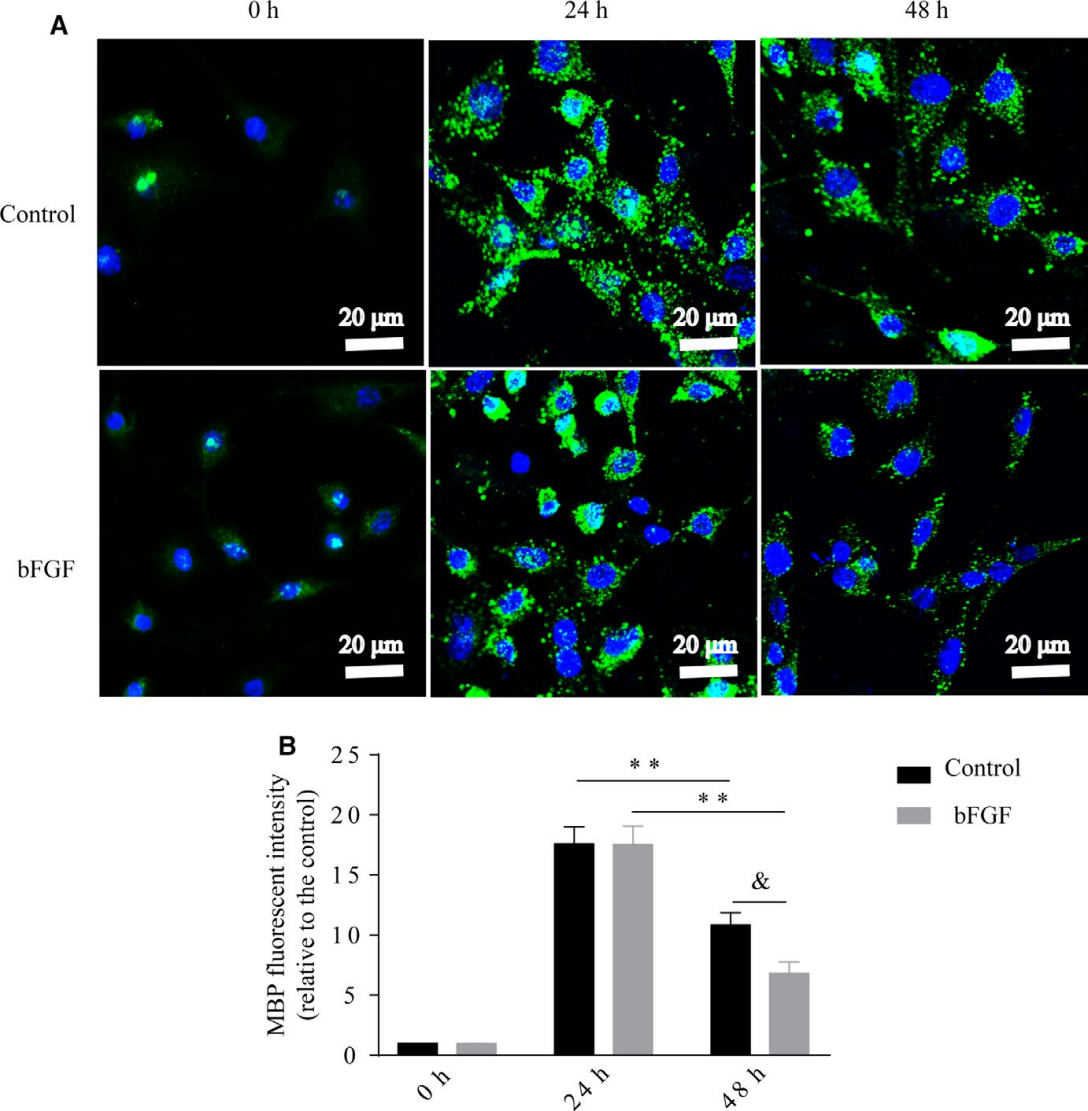
bFGF promotes myelin clearance by SCs in vitro. RSC 96 cells were exposed to homogenized myelin debris for 24 h. Then, 100 ng mL^‐1^ bFGF was added to the culture medium for incubating another 24 h. The myelin debris existed within the SCs at indicated time were immunostaining for MBP^+^ shown in (A). Corresponding quantification of MBP^+^ fluorescent intensity were shown in (B). Data are presented as means ± SEM. bFGF, basic fibroblast growth factor; SC, Schwann cell. Control_24h_ vs Control_48h_: ^**^ *P* < 0.01; bFGF_24h_ vs bFGF_48h_: **P < 0.01; Control_48h_ vs bFGF_48h_: ^&^ *P* < 0.05

To test the role of bFGF on regulating SCs to remove myelin debris, we conducted SC phagocytosis and clearance of myelin in vitro, that is incubating a SC line, RSC 96 cells, with myelin debris for 24 hours, then adding with or without bFGF to the medium for incubating another 24 hours (recording as 48 hours). Myelin debris were obtained from homogenized nerve tissue, which were labelled with MBP signal. We found MBP intensity was weak prior to incubation with myelin debris (0 hour). After stimulating in vitro with myelin debris for 24 hours, MBP + puncta were engulfed and accumulated within the SC line. But this circumstance was substantially altered in the following time. We found the myelin accumulation within the SCs was largely reduced at culturing time of 48 hours, especially for the bFGF‐treating group (Figure 4A). The quantitative result in Figure 4B showed that the ratio of total MBP^+^ fluorescence density in control group: bFGF group was near twofold, suggesting bFGF has significant effect in mediating myelin clearance by SCs.

### 3.5 bFGF‐induced autophagy activation is responsible for myelin debris clearance

**FIGURE 5:**
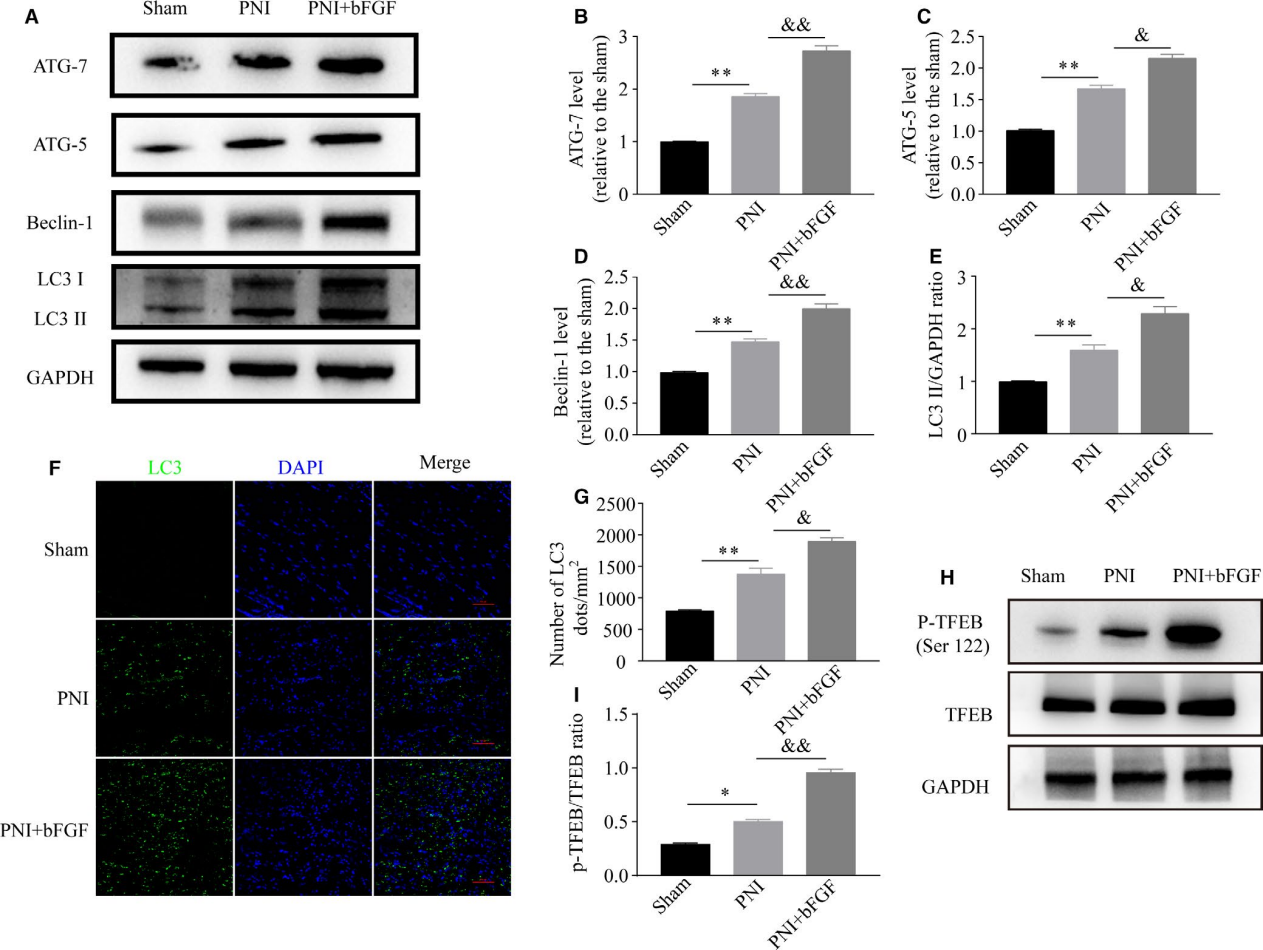
bFGF up‐regulates the autophagy response through TFEB signalling after PNI for 5 d. A, The representative images of Western blots for autophagy markers: Atg5, Atg7, Beclin‐1 and LC3 in sciatic nerves of sham, PNI and PNI + bFGF rats. B‐E, The blot densitometrical analyses of above proteins. F, Representative images of immunostaining for LC3 in the sham, PNI and PNI + bFGF groups at day 5 post‐injury. Scale bar = 100 μm. G, Statistical analysis of the postive LC3 dots in each group. H and I, Representative immunoblotting images and quantitative ratio of *p*‐TFEB/TFEB in each group. Values are means ± SEM. bFGF, basic fibroblast growth factor; PNI, peripheral nerve injury; TFEB, transcription factor EB. *P < 0.05, **P < 0.01 as compared to the control group. ^&^ *P* < 0.05, ^&&^ *P* < 0.01 as compared to the PNI group

To evaluate whether autophagy activation is required for the action of bFGF on myelin cleavage during WD, we analysed the expressing changes of the essential autophagy proteins, including ATG‐7, ATG‐5, Beclin‐1 and LC3, in sham, PNI and PNI + bFGF groups after 5 day post‐surgery via Western blotting. The result showed that these autophagy biomarkers were all expressed at low levels in the normal condition, but were significant up‐regulation following PNI. Administration of bFGF could further increase this trend, manifesting the highest level among these three groups (Figure 5A‐E). Similarly, the trend of LC3 immunoreactivity was in accordance with the immunobloting results, that is PNI + bFGF group > PNI group > sham group (Figure 5F,G). All of these results suggest bFGF‐medicated myelin clearance is closely associated with autophagy activation during WD.

Next, we further reveal the mechanism of bFGF‐induced autophagy activation on regulating myelin clearance. Previous studied had regarded transcription factor EB (TFEB) as a global controller for regulating lysosomal biogenesis and autophagy,^30^ and we attempted to explore this relationship. Using immunoblotting, we detected the changes of TFEB expression in sciatic nerves after 5 days post‐injury. The result showed that the phosphorylation of TFEB was significantly increased after PNI, but bFGF treatment could further enhance this trend (Figure 5H). Quantitative data also revealed that the ratio of P‐TEFB/TEFB in PNI + bFGF groups was as high as approximately two times that of the PNI group (Figure 5I, *P* < .01). These results indicate that bFGF‐medicated autophagy activation is closely associated with targeting TFEB pathway.

### 3.6 bFGF‐medicated autophagy activation via maintaining the fluent autophagic flux

**FIGURE 6:**
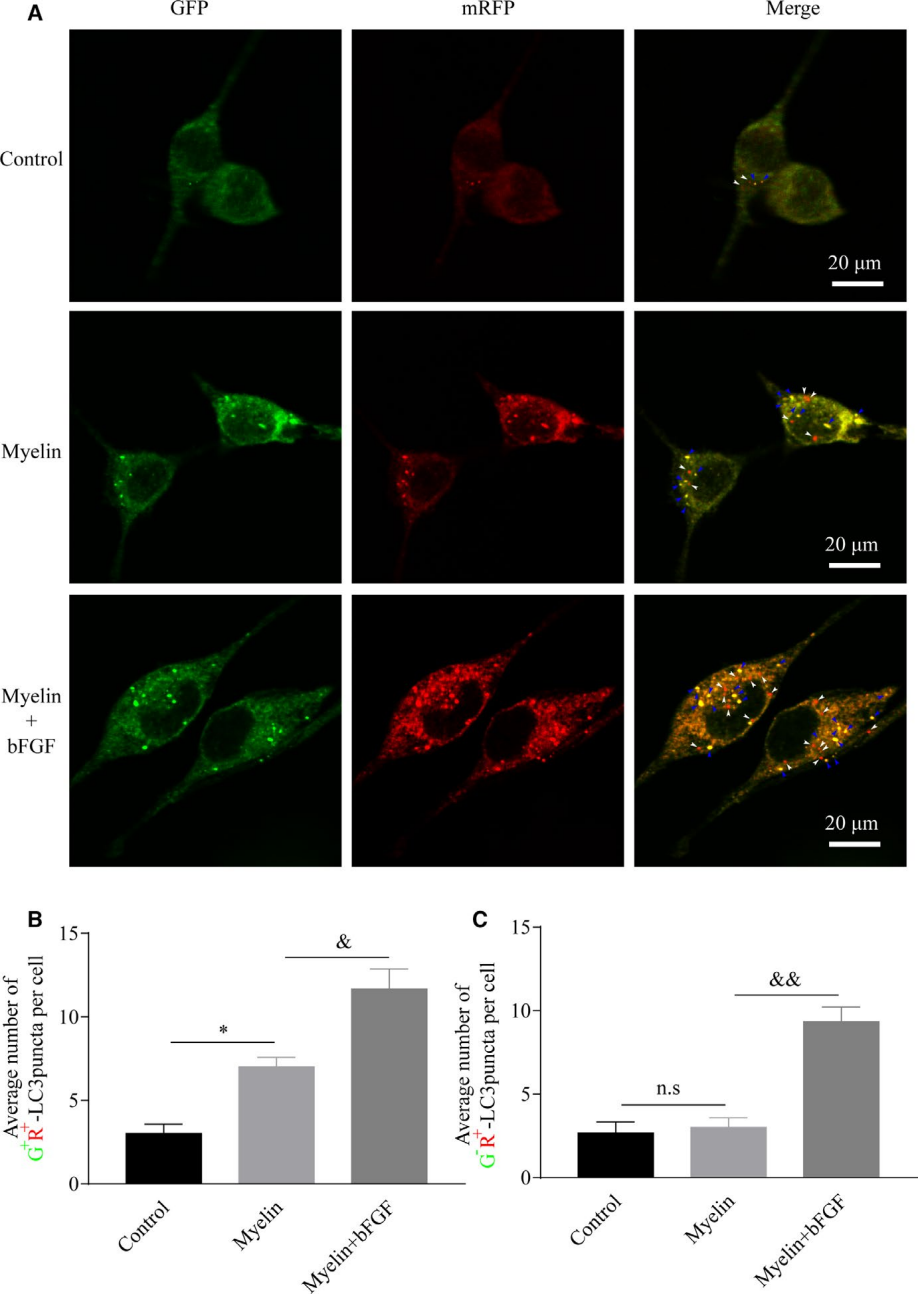
bFGF‐induced autophagic activation is through maintaining autophagic flux's flow. A, Representative images of SCs were infected with mRFP‐GFP‐LC3 vectors and imaged by laser‐scanning confocal microscopy. The Autophagosomes and autophagolysosomes were identified by blue arrowheads and white arrowheads. Scale bar = 20 μm. B and C, quantification of the average number of G^+^R^+^‐LC3 (autophagosome) or G^‐^R^+^‐LC3 (autolysosome) dots for each cell. The data are represented as means ± SEM. n.s representing compared groups were no significant difference. bFGF, basic fibroblast growth factor; PNI, peripheral nerve injury; SC, Schwann cell. *P < 0.05 as compared to the control group. ^&^ *P* < 0.05; ^&&^ *P* < 0.01 as compared to the PNI group

Autophagic flux enhancement or blockade can increase the level of autophagy‐related proteins expression, but result contrary biological effects.^22^, ^31^ The increased autophagosomes fusing with lysosomes, that is autophagic flux enhancement, is contributed to cellular debris degradation and neuronal development. By contrary, if the interdiction in fusion of autophagosomes with lysosomes occurs, termed autophagic flux blockade, this will lead to metabolic imbalance and axonal atrophy. To distinguish the difference of autophagy activation mentioned above, we treated SC line with homogenized nerve segments in combination with/without bFGF for 12 hours and monitored autophagic flux changes using a tandem mRFP‐GFP‐LC3 probe. Immunofluorescence and quantitative analyses of the number of RFP^+^/GFP^+^ dots (ie autophagosome) were aggregated within the cytoplasm in both myelin culturing groups, especially for the bFGF‐treating cells (Figure 6A). For the present of RFP^+^/GFP^‐^ (ie autolysosome) dots, the myelin + bFGF group had higher number of RFP^+^/GFP^−^ dots when compared to the other groups without bFGF treatment which displayed no statistical significance (Figure 6B,C). These data imply that the activation of autophagy after PNI is attribute to the obstacle of autophagosome fusing with lysosome, whereas bFGF can maintain fluent autophagic flux to enhance autophagy level within SCs.

## 4 DISCUSSION

In the present study, we used a rat PNI model in vivo and myelin phagocytosis by SCs in vitro to reveal the cellular and molecular mechanisms for bFGF modulating early nerve regeneration and the role of exogenous bFGF in regulating myelin removal during WD. Histological and biochemical results indicated that administration of bFGF at optimized doses indeed promoted axonal regrowth and myelination at early 14 days post‐injury. Interestingly, this beneficial effects of bFGF on promoting nerve recovery were mainly attributed to accelerate the phagocytosis of degenerating myelin within SCs, which was probably regulated by TEFB‐medicated autophagy activation. Together, these results highlight the important role of autophagy‐medicated myelin debris clearance on the repair of PNI.

The PNS has a certain regenerative potentiality after nerve injury. A prerequisite for successful regeneration is effective and rapid clearance of myelin debris performed mainly by SCs and macrophages in damaged nerve stump.^6^, ^32^, ^33^ During this period, the expression of multiple growth factors showed a temporal and spatial manner, but their secretion was not satisfied the demand for complete axon regeneration and function recovery.^34^ As a typical representative, bFGF has a remarkable capacity for regulating various cellular behaviours, including cell survival, proliferation and differentiation.^35^ In the previous study, the major reason of bFGF for promoting peripheral nerve restoration were focus on the following aspects: (a) maintaining neural survival and suppressing their apoptosis^36^; (b) facilitating axonal outgrowth and remyelination^37^; (c) stimulating angiogenesis and vessel remodelling^38^; and (d) inhibiting glial scar formation.^39^ However, up to now, no evidence has explored the relationship between nerve regeneration and damaged myelin clearance after administration of exogenous bFGF for treating PNI. Presently, we observed that redundant myelin fragment accumulation at 5 day post‐injury was followed by scarce nerve regeneration and thin myelin formation at day 14 post‐injury. Moreover, experimental data in vivo and in vitro further revealed the reason of this effect was closely associated with bFGF‐medicated myelin phagocytosis, which selectively targeted SCs digestion, highlighting bFGF‐induced myelin clearance in SCs was essential for supporting nerve regrowth at the early stage of PNI.

We further established that bFGF–mediated myelin debris clearance was driven by profound activation of autophagy, an intracellular catabolic process for eliminating damaged organelles and altered proteins. In the pathology of traumatic PNI, autophagy has recently been regarded as a potential molecular mechanism for the bulk removal of damaged myelin by SCs.^10^, ^18^ Accumulating evidence had demonstrated that activating autophagy in nerves system was shown to prevent neurodegeneration, axonal atrophy and microtubule disorder.^23^, ^40^, ^41^ Hence, the role of autophagy in neuroprotection has opened an alternative therapeutic option in repairing PNI. In this study, we found that the elevated autophagic hallmarks had been detected following PNI, but the level was inferior to that administration of bFGF treatment. Hence, bFGF‐mediated autophagy enhancement is contributed to myelin clearance during WD.

Extensive autophagic activity does not always mean a fluent autophagic flux, because the induction of autophagy activity depends on two aspects, either the rate of autolysosome formation exceeding their degradation, or retention of the autolysosome degradation pathway.^42^, ^43^ To reveal which pattern controlling bFGF‐medicated autophagy activation after PNI, we monitored autophagic flux through transfection of adenovirus harbouring GFP‐mRFP‐LC3. Our results showed that bFGF increased autophagosome fusion with the lysosome to correct the disorganized autophagy process in vitro. This demonstrated that bFGF enhances autophagy activation through autophagosomes fusing with lysosomes to maintain fluent autophagic flux. It should be noted that lysosomal enzymolysis may also participate in myelin degradation. It had been shown that autophagy was an intracellular lysosome‐dependent pathway for degrading degenerative debris^44^ and lysosomal proteases had their intrinsic capability for degrading these waster during the stage of autophagosomes fusing with lysosomes.^45^ Moreover, disturbance of lysosomal bioactivity could increase the pathological change of autophagy flux following the central nervous injury.^46^ Based on these facts, we deem that bFGF can enhance lysosome function, induce autophagy activation and maintain the fluent autophagy flux in SCs to degrade myelin debris.

Besides, we also demonstrated that bFGF‐induced autophagy activation and lysosome function is probably through targeting TFEB pathway. However, we cannot clarify the issue on bFGF‐induced TFEB phosphorylation to activate autophagy through direct or indirect way. After searching extensively, we understand TFEB‐medicated autophagy activation and lysosomal function is tightly linked to other transcription factors. For instance, previous studies had demonstrated that TFEB could increase the expression of autophagy‐lysosomal genes and enhance cellular clearance and metabolism by directly binding to CLEAR signalling.^30^, ^47^ Recently, studies also revealed that TFEB also interacted with STIP1 homology and U‐Box containing protein 1 (STUB1) to rescue autophagy disorder and maintain the homeostasis of mitochondrial biogenesis.^48^ Additionally, TFEB subcellular localization and activity is also tremendously affected by mTOR‐dependent hierarchical multisite phosphorylation.^49^ Thus, we speculated that bFGF‐medicated TFEB signalling activation was likely regulated by CLEAR, STUB1 or mTOR, but the exact mechanism need explore in further.

In conclusion, this report illustrates the acceleration of myelin fragments clearance is necessary for bFGF exerting its biofunction on repairing early nerve repair, which can be regulated by TFEB‐medicated autophagy activation within SCs. Further studies on identifying the exact mechanism of different GFs regulating myelin clearance may potentially provide novel targets for restoring damaged nerve structure and function.

## CONFLICT OF INTEREST

The authors declare that they have no conflicts of interest.

## AUTHOR CONTRIBUTIONS

**Yongsheng Jiang:** Conceptualization (equal); Data curation (equal); Formal analysis (equal); Funding acquisition (equal); Methodology (equal). **Jiahong Liang:** Conceptualization (equal); Data curation (equal); Formal analysis (equal); Investigation (equal); Methodology (equal). **Rui Li:** Conceptualization (equal); Data curation (equal); Formal analysis (equal); Investigation (equal); Writing‐original draft (equal). **Yan Peng:** Data curation (equal); Formal analysis (equal); Investigation (equal). **Jiangli Huang:** Data curation (equal); Validation (equal); Visualization (equal). **Lijiang Huang:** Funding acquisition (lead); Writing‐review & editing (lead).

## Supporting information

Supplementary Material
