# Supplementary material for: Basic fibroblast growth factor accelerates myelin debris clearance through activating autophagy to facilitate early peripheral nerve regeneration
Source: J Cell Mol Med. 2021 Jan 29;25(5):2596–608. doi: 10.1111/jcmm.16274 (PMC7933946; doi:10.1111/jcmm.16274)
Supplement: Supplementary file 1 — Supplementary Material [file JCMM-25-2596-s001.docx]

**Lysosomal PH detection.** SCs culturing in myelin debris+bFGF at indicated time points was added 5 μM 2-(4-pyridyl)-5-((4-(2-dimethylaminoethyl-aminocarbamoyl)

methoxy)phenyl)oxazole (PDMPO) probe (40768ES50, Yeasen, China) at 37℃ for 5 min. After being washed twice with PBS, the fluorescent intensity of cells in different groups was acquired by an inverted microscope (dichroic is 400 nm, emitter is BP490-530 nm for green images and BP417-483 nm for blue images). The fluorescence emission intensity ratio of blue/green in the lysosomal region was calculated and processed according to the procedure of Chen *et al* [^1^](#_ENREF_1).

**The red fluorescent intensity of pHrodo-labeled myelin debris at different PH condition.** Saline was added 0.01M HCl to adjust PH with the value of 3.7, 4.1 and 4.5 using PH meter. Then, the prepared pHrodo-labeled myelin debris were supplemented into saline to acquire the final concentration of 800 μg mL^-1^ and incubated at 37℃ for 0.5 h. Images were taken using an inverted microscope (Nikon, Japan).

Fig. S1


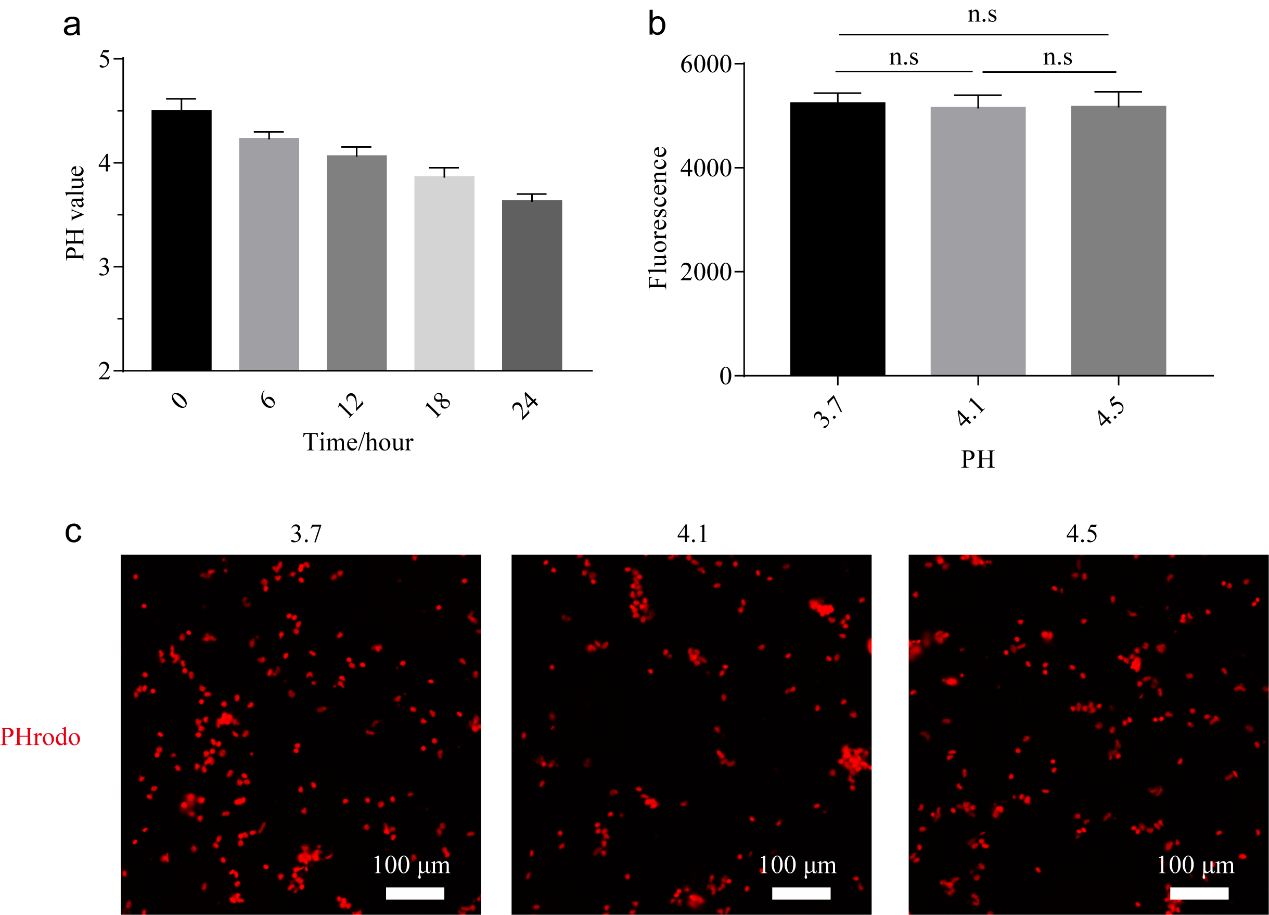


**Fig. S1 bFGF accelerating SC engulfment is not through enhancing lysosome acidification.** (a) Measurement of lysosomal pH during SCs engulfing the labeled debris within 24 h (6-hour interval) stimulated by bFGF using PDMPO probe. (b, c) The red fluorescence images of pHrodo-labeled myelin debris immersed in saline with the PH values of 3.7, 4.1 and 4.5. Scable bar = 100 μm. Data are shown as means ± SEM. n.s represents not significant difference between the compared groups.

**Reference**

1. **Chen CS, Martin OC, Pagano RE.** Changes in the spectral properties of a plasma membrane lipid analog during the first seconds of endocytosis in living cells. *Biophys J*. 1997; 72: 37-50.
